# Longitudinal genotype-phenotype analysis in 86 patients with *PAX6*-related aniridia

**DOI:** 10.1172/jci.insight.148406

**Published:** 2021-07-22

**Authors:** Vivienne Kit, Dulce Lima Cunha, Ahmed M. Hagag, Mariya Moosajee

**Affiliations:** 1 Moorfields Eye Hospital, NHS Foundation Trust, London, United Kingdom.; 2 UCL Institute of Ophthalmology, London, United Kingdom.; 3 Great Ormond Street Hospital for Children, NHS Foundation Trust, London, United Kingdom.; 4 The Francis Crick Institute, London, United Kingdom.

**Keywords:** Genetics, Ophthalmology, Genetic diseases

## Abstract

Aniridia is most commonly caused by haploinsufficiency of the *PAX6* gene, characterized by variable iris and foveal hypoplasia, nystagmus, cataracts, glaucoma, and aniridia-related keratopathy (ARK). Genotype-phenotype correlations have previously been described; however, detailed longitudinal studies of aniridia are less commonly reported. We identified 86 patients from 62 unrelated families with molecularly confirmed heterozygous *PAX6* variants from a UK-based single-center ocular genetics service. They were categorized into mutation groups, and a retrospective review of clinical characteristics (ocular and systemic) from baseline to most recent was recorded. One hundred and seventy-two eyes were evaluated, with a mean follow-up period of 16.3 ± 12.7 years. Nystagmus was recorded in 87.2% of the eyes, and foveal hypoplasia was found in 75%. Cataracts were diagnosed in 70.3%, glaucoma in 20.6%, and ARK in 68.6% of eyes. Prevalence, age of diagnosis and surgical intervention, and need for surgical intervention varied among mutation groups. Overall, the missense mutation subgroup had the mildest phenotype, and surgically naive eyes maintained better visual acuity. Systemic evaluation identified type 2 diabetes in 12.8% of the study group, which is twice the UK prevalence. This is the largest longitudinal study of aniridia in the UK, and as such, it can provide insights into prognostic indicators for patients and guiding clinical management of both ocular and systemic features.

## Introduction

Aniridia (OMIM 106210) is a rare congenital panocular condition, with an incidence of 1:40,000 to 1:100,000, that demonstrates no predilection for race or sex (1, 2). It is inherited autosomal dominantly with high penetrance, although there is significant intra- and interfamily phenotypic variability, and one-third of cases are sporadic (2). Typical aniridia is characterized by variable iris hypoplasia, which can present with complete absence of the iris (or only a small iris stump seen on gonioscopy), partial iris defects, and, in some instances, a full iris with only abnormal surface architecture or transillumination defects (3, 4). Patients typically have impaired visual acuity (0.70–1.00 logarithm of the minimum angle of resolution [logMAR]) and nystagmus from birth (1, 2), attributable to foveal hypoplasia (3, 5). The development of cataracts, glaucoma, and aniridia-related keratopathy (ARK) is common and further contributes to a progressive deterioration in vision (1, 2, 6–8).

Aniridia is caused by heterozygous mutations involving the paired box 6 (*PAX6*) gene, which is considered the master regulator of the eye (9, 10). *PAX6* (OMIM 607108) encodes a transcription factor that is highly expressed throughout the developing eye and postnatally in the cornea, conjunctiva, iris, ciliary body, lens, and retina (11, 12). Typical aniridia phenotypes are usually associated with loss-of-function heterozygous *PAX6* mutations, which result in haploinsufficiency (13–17), with nearly 70% (i.e., nonsense, frameshift from insertion-deletion, and most intronic/splicing variants) leading to the introduction of a premature termination codon (PTC). Other variants causing *PAX6* haploinsufficiency include whole-gene deletions or mutations in the *PAX6* 3′ regulatory region (18, 19). Mutations leading to the C-terminal extension (CTE) of *PAX6* protein are less common, with some reports linking these variants to more severe aniridia phenotypes, comparable to PTCs (3, 20, 21). In contrast, missense *PAX6* variants are usually linked to milder aniridia cases or, more frequently, to nonaniridia-related phenotypes, such as microphthalmia and ocular coloboma (OMIM 120430 and 120200) or foveal hypoplasia 1 (OMIM 136520; refs. 18, 22–24).

Outside of the eye, *PAX6* expression plays a vital role in the embryological development of the brain (25, 26) and pancreas (27), and postnatally, it influences the expression and secretion of various pancreatic derived hormones, including insulin (27). Systemically, isolated *PAX6* mutations have been associated with reports of type 1 and 2 diabetes (25, 26, 28), obesity (28), brain anatomical and neurodevelopmental anomalies, neurobehavioral disorders, and autism spectrum disorder (29–35). Furthermore, aniridia is also observed in syndromes with systemic involvement, which include WAGR syndrome (Wilms tumor, aniridia, genitourinary anomalies, and mental retardation, OMIM 194072) and the closely related WAGRO syndrome (Wilms tumor, aniridia, genitourinary anomalies, mental retardation, and obesity, OMIM 612469; ref. 36). Both conditions involve deletions of the *PAX6* gene as well as *WT1* (WAGR) or *WT1* and *BDNF* (WAGRO; ref. 18).

The spectrum of PAX6 mutations and clinical phenotypes of patients with aniridia have been well described (3, 7, 8, 37–42), and most studies have reported no clear genotype-phenotype correlations (8). More recent studies report cross-sectional data but do not map disease progression (7, 39). Hence, the purpose of our study is to examine the longitudinal natural history of aniridia, with a focus on genotype-phenotype correlations in a large cohort of molecularly confirmed patients with *PAX6*-related aniridia.

## Results

### Molecular and clinical characteristics of patients.

One hundred and seventy-two eyes from 86 individuals (52 female and 34 male participants) were evaluated in this study. There were 62 families in total, including 60 patients from 36 families with familial aniridia, 18 patients with sporadic aniridia with de novo *PAX6* pathogenic variants, and 8 individuals in which the family history was not documented. Ethnicity was not documented in 41.9% of individuals; however, 48.8% were White (*n* = 42), 3.5% were Black (*n* = 3), and 5.8% were Asian (*n* = 5). The age at baseline visit ranged from 1 month to 66 years (mean 15.7 ± 16.0 years). The age at final visit ranged from 1 year to 71 years (mean 31.7 ± 18.1 years, excluding 4 patients with a single visit). Detailed genotype and phenotype data for all patients are provided in Supplemental Table 1 (supplemental material available online with this article; https://doi.org/10.1172/jci.insight.148406DS1), and a summary of patient demographics and clinical characteristics is presented in Table 1.

### Spectrum of heterozygous PAX6 variants.

**Figure 1:**
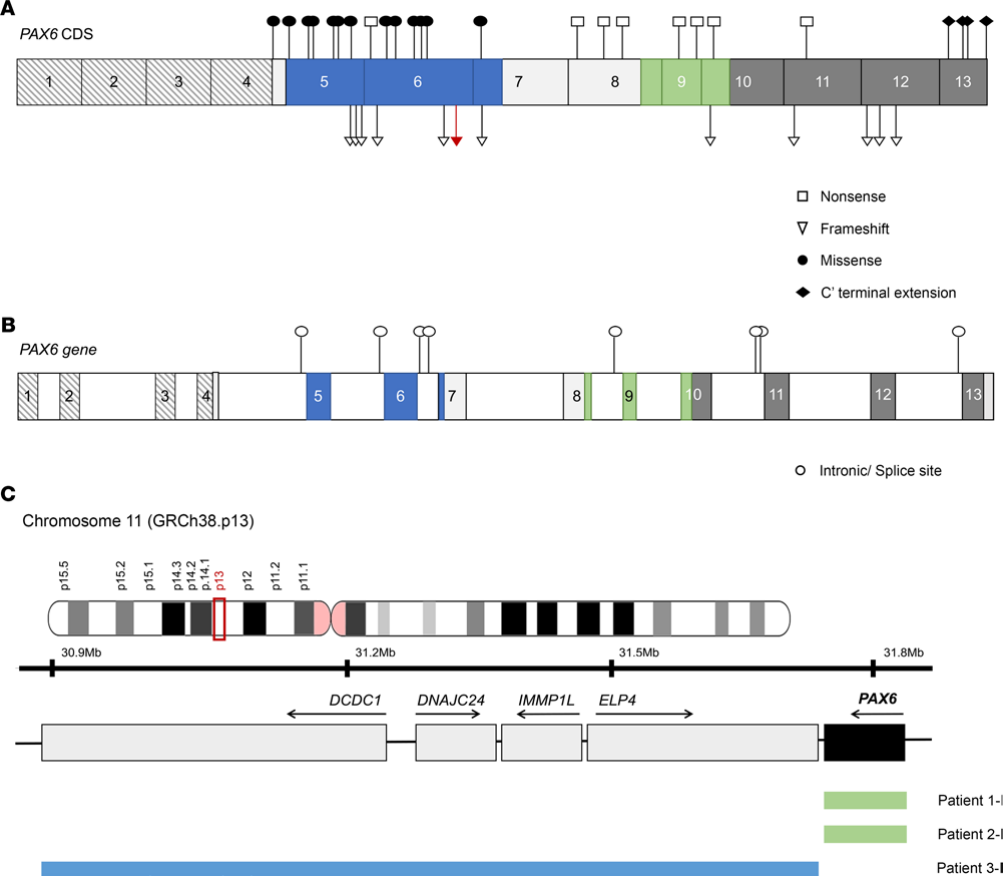
Distribution of the 48 different *PAX6* variants identified in this cohort. (**A**) *PAX6* coding sequence (CDS) is shown with numbered exons and colors representing the respective protein domains: paired domain (blue, exons 5–7), homeodomain (green, exons 8–10), and proline-serine-threonine–rich domain (dark gray, exons 10–13). Linker region is represented in light gray (exons 7–8). Striped boxes represent noncoding exons 1–4. Variants are represented by white squares (nonsense), white triangles (frameshift), black circles (missense), and black diamonds (C-terminal extension). The previously undescribed variant is highlighted in red. (**B**) Schematic of *PAX6* including intronic regions (white boxes) was used to show distribution of intronic/splice site variants, represented as white circles. Both schematics represent *PAX6* transcript NM_000280.4 encoding protein isoform NP_000271.1. (**C**) Schematic representation of deletions in 11p13 encompassing either whole *PAX6* gene or the regulatory regions 3′ of *PAX6* in the *ELP4* gene. *PAX6* is highlighted in black and neighbor genes are represented by gray boxes. Colored bars represent approximate coordinates of deletions identified in 3 patients in this study (patients 1-i, 2-i, and 3-i). The exact chromosomal coordinates were not obtained from the genetic screening service. Adapted from ref. 18.

Detailed genetic results for all 86 patients included in this study are presented in Supplemental Table 1. Patients were divided into 6 groups according to the type of *PAX6* variants identified; nonsense (14 of 86, 16.3%), frameshift (21 of 86, 24.4%), intronic/splice site (15 of 86, 17.4%), CTE (14 of 86, 16.3%), and missense (19 of 86, 22.1%) groups (Figure 1, A and B). *PAX6* whole-gene deletions were identified in 2 patients (2.4%), and a larger deletion encompassing the *PAX6* 3′ regulatory region in the *ELP4* gene was detected in 1 patient (1.2%) (Figure 1C); these patients were grouped into the gene deletion category.

There were 48 different variants identified in this cohort, with only one previously undescribed frameshift variant, c.345_351dupTAACATA p.(Pro118*), which occurred sporadically in one patient (patient 23-i) presenting with bilateral complete iris hypoplasia, foveal hypoplasia, and cataracts (Supplemental Table 1). The majority of variants were located in exons 5 (9 in total; 3 frameshift and 6 missense), 6 (9 in total; 1 nonsense, 3 frameshift, and 5 missense), 9 (2 nonsense), and 13 (4 CTE; Figure 1A). The majority of intronic variants were also located in intron 6 (Figure 1B). Variants causing the introduction of a PTC were distributed across the gene, while all CTEs were located in exon 13. As supported by other cohort studies, missense variants are exclusively located in exons 5 and 6, which code for the paired domain of the PAX6 protein. The exception is patient 50-I, who was identified with a change in the start codon in exon 4, c.2T>G, p.(Met1Arg; ref. 43). This variant is predicted to cause failure of translation or initiation from cryptic sites, but due to the amino acid change (Met to Arg) it was classified into the missense group.

Patient 21-I has the variant c.174C>T in exon 6, which is predicted in silico to be synonymous (p.[Gly58 = ]). However, functional characterization of this variant showed that it leads to exon 6 shortening by introducing a new donor site and consequent altering the reading frame, resulting in the formation of a PTC (p.[Arg59Valfs*12]) and likely transcript degradation by nonsense-mediated decay (44, 45); hence, it was classified as a frameshift. Patient 47-I was identified with 2 independent *PAX6* variants: intronic variant c.917-1G>C and missense variant c.1112C>A, p.(Thr371Asn; ref. 20). However, the missense change was predicted as benign by in silico tools (data not shown), classifying this patient into the intronic variant group.

### Longitudinal changes in visual acuity.

**Figure 2:**
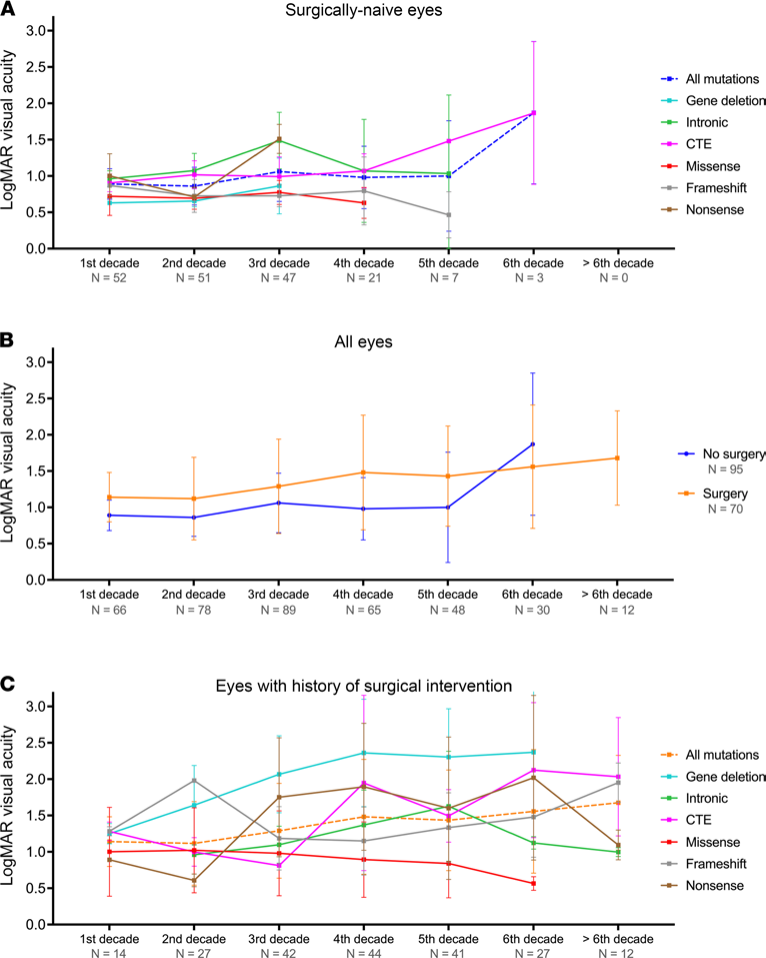
Best-corrected visual acuity in patients with *PAX6* mutations. Line charts demonstrate the longitudinal change in best-corrected visual acuity in this cohort in (**A**) different mutation groups with surgically naive eyes, (**B**) all eyes with or without surgical intervention, and (**C**) comparing mutation groups with a history of surgical intervention. Data represent mean ± SD. LogMAR, logarithm of the minimum angle of resolution, CTE, C-terminal extension.

Longitudinal changes in best-corrected visual acuity (BCVA) were assessed across decades to map the natural history, and the cohort was divided into subgroups based on the type of mutation and history of surgical intervention (for the following ocular indications: cataract, glaucoma, and ARK; Figure 2). A total of 165 eyes from 83 patients were included in the BCVA analysis. Ninety-five eyes from fifty patients did not have a history of surgical intervention (i.e., surgically naive eyes), whereas the remaining 70 eyes from 38 patients underwent at least 1 ocular surgical procedure. In the surgically naive eyes, BCVA in the first decade of life was 0.85 ± 0.14 logMAR (mean ± SD), with a progressive decline in visual acuity observed over the following decades. Compared against the mean of all mutations, patients with intronic and CTE variants trended toward a worse visual outcome with increasing age, while patients with gene deletions and frameshift and missense mutations maintained stable visual acuity over time (Figure 2A). For the first 5 decades, surgically naive eyes maintained a mean BCVA logMAR of 1.0, compared with those having undergone surgical intervention who had a mean BCVA of more than 1.5 logMAR (Figure 2B). Although the BCVA was shown to worsen in the 6th decade in the surgically naive group, which was composed of patients who only harbored CTE variants and, therefore, not an accurate comparison. Among eyes that had undergone surgical intervention, only the missense group maintained a better visual acuity than the overall mean. All other mutation subgroups demonstrated a trend toward a gradual decline in visual acuity from the third decade onward (Figure 2C), except for the gene deletion group, which had the most progressive visual decline from the first decade (Figure 2C). The gene deletion group is small (4 eyes from 2 patients), and therefore, the visual acuity trends observed for gene deletions is only reflective of observed natural history of our cohort. A larger number of patients with gene deletions will be required for future studies.

### Nystagmus and degree of foveal hypoplasia varies with mutation subgroup.

Nystagmus was recorded in 75 of 86 patients (87.2%), and foveal hypoplasia was documented in 129 of 172 eyes (75%). Of the remaining patients, we excluded those without recorded nystagmus or fovea hypoplasia from further analysis. Spectral domain optical coherence tomography macula scans were available for 29 eyes from 17 patients to classify foveal hypoplasia into mild (grades 1 and 2) and severe (grades 3 and 4). The grade of foveal hypoplasia was symmetrical between both eyes of each patient where both OCT images were available. There was a significant difference in severity between mutation groups (*P <* 0.001) with intronic (*Z* score = 2.2) and frameshift (*Z* score = 2.0) subgroups displaying a more severe foveal hypoplasia, while the missense (*Z* score = –4.0) subgroup presented milder grades (Table 2).

Of 29 eyes from 17 patients used for foveal hypoplasia grading analysis, only 4 eyes from 2 individuals were from the same family, of which all demonstrated the same grade of foveal hypoplasia. Due to the small number, further analysis could not be performed on intrafamilial variability. Spearman’s correlation analysis was performed on foveal hypoplasia grade and first recorded visual acuity. This demonstrated a significant positive correlation between higher foveal hypoplasia grade and worsening visual acuity (*rho* = 0.46, *P =* 0.012).

### Spectrum of iris hypoplasia varies with PAX6-specific mutation subgroups.

Iris abnormalities were reported in 154 of 172 eyes (89.5%). The majority displayed ocular symmetry; however, asymmetric iris hypoplasia was observed in 3 patients from the frameshift (19-I), CTE (30-I), and missense (62-I) mutation groups. Fifty-two eyes had grade 6 complete iris hypoplasia (30.2%), with higher prevalence in nonsense (42.9%), intronic (46.7%), and gene deletion (33.3%) subgroups; this was found at a lower prevalence in the missense (18.4%) and CTE (17.9%) groups. Conversely, partial iris hypoplasia (grades 1–5) was seen in 65 eyes and was more prevalent in the missense (50%) and CTE (53.6%) subgroups, with lower prevalence of nonsense (7.1%), intronic (33.3%), and gene deletion (16.7%) patients (Table 1). Eighteen eyes (10.5%) had normal iris architecture with no structural abnormalities documented; these were observed in the missense (26.3%), CTE (14.3%), and frameshift (9.5%) mutation subgroups. Thirty-six eyes had documented iris abnormalities, but the degree was not recorded (20.9%). For severity analysis, grades 1–4 were considered mild, and grades 5–6 were considered severe. Overall, the missense mutation group had significantly milder (*Z* score = –3.5) grades of iris hypoplasia compared with the nonsense (*Z* score = 4.0), frameshift (*Z* score = 2.7), intronic (*Z* score = 2.7), and gene deletion (*Z* score = 2.0) groups, which demonstrated significantly higher grades of iris hypoplasia (Table 2).

Within this cohort, iris hypoplasia grade was fully documented for 10 families, which consisted of 2 affected members. In 4 families, there was no intrafamilial variability relating to iris hypoplasia (families 18, 25, 45, and 59). Seven families (families 19, 27, 31, 34, 55, 58, and 60) demonstrated mild intrafamilial variability (no iris abnormality to mild iris hypoplasia, or severe grades of aniridia only), and 1 family (family 42) showed more substantial differences, ranging from mild grade 1 to severe grade 6 iris hypoplasia.

### Natural history of cataracts in patients with PAX6 mutations.

**Figure 3:**
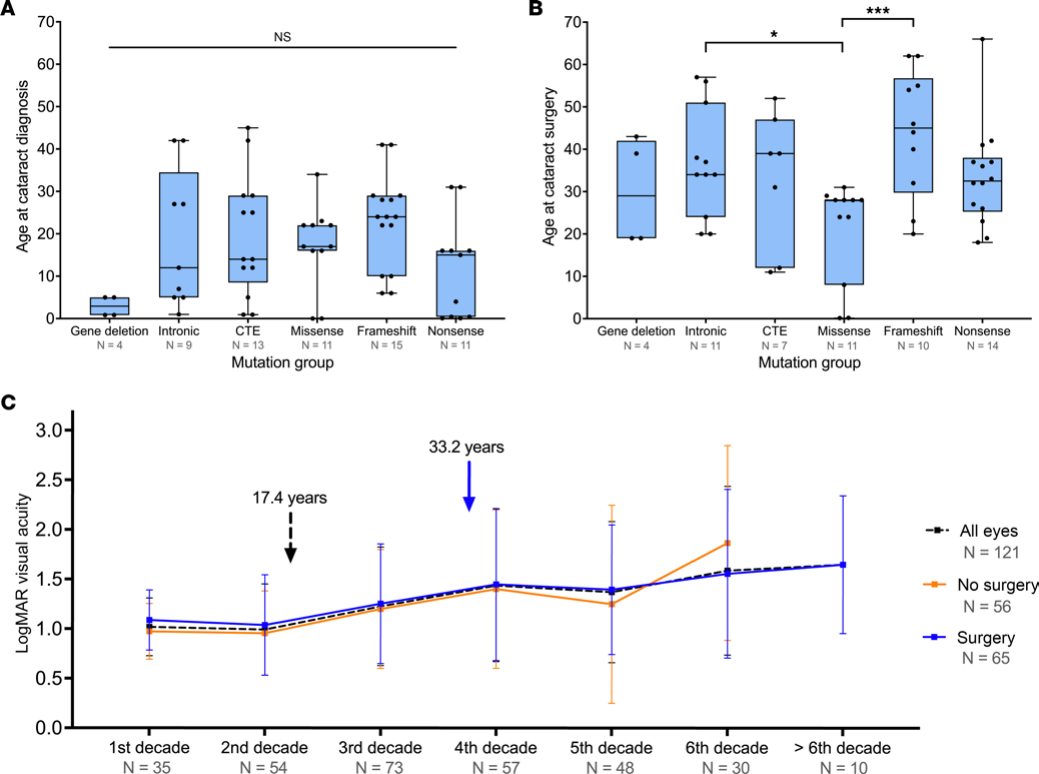
Patients with *PAX6* mutations and cataracts. Box-and-whisker plots represent the differences in (**A**) age at diagnosis and (**B**) age at surgery among mutation groups. The blue box represents the 25th to 75th percentiles. The black line within the box represents the median. Whiskers extend to the minimum and maximum values. All data points were superimposed on the graph. Kruskal-Wallis and Dunn’s post hoc tests were used to compare among mutation groups. (**C**) Line graph demonstrates the change in logarithm of the minimum angle of resolution (LogMAR) visual acuity in patients with cataracts. Data represent mean ± SD. Black dashed arrow and blue arrow represent the mean age at cataract diagnosis and surgery, respectively. CTE, C-terminal extension. *P < 0.05, ***P < 0.001.

Cataracts were diagnosed in 121 of 172 eyes (70.3%) from 62 individuals (59 bilateral and 3 unilateral, 23 male and 39 female patients). The mean age of cataract diagnosis was 17.4 ± 12.9 years. No statistically significant difference was detected in the age of onset between different mutation subgroups (*P =* 0.053; Figure 3A), and sex had no role in the prevalence (*P =* 0.12). However, the prevalence varied significantly (*P =* 0.047), with missense and frameshift mutations exhibiting a lower prevalence (57.9%, *Z* score = –1.9 and 59.5%, *Z* score = –1.8, respectively) compared with other groups (Table 2). Cataract surgery was performed in 65 eyes (53.7%, 29 bilateral and 7 unilateral, 3 male and 43 female patients) at a mean age of 33.2 ± 14.7 years. Patient age at the time of cataract operation was statistically different between groups (*P =* 0.029); the mean age in the missense subgroup was significantly younger (20.8 years) compared with that of individuals in the frameshift (43.8 years, *P =* 0.001) and intronic (36.8 years, *P =* 0.014) mutations (Figure 3B). Comparing mean visual acuity of operated and unoperated eyes, there is no trend toward improvement in visual acuity in the operated group (Figure 3C).

### Natural history of glaucoma in patients with PAX6 mutations.

In total there were 37 of 172 eyes with recorded glaucoma; however, 2 eyes (from 2 different patients) had secondary glaucoma following surgical intervention and were excluded from further statistical analysis. (a) Patient 53-I is a White female patient who had undergone cataract surgery at 8 and 10 weeks of age for right and left cataracts, respectively, and subsequently developed secondary glaucoma requiring cyclodiode laser to the left eye at 15 months of age, and topical glaucoma treatment is currently used in both eyes (although the right eye maintained a healthy cup: disc ratio of 0.2). (b) Patient 46-III, of unknown ethnicity, developed glaucoma 16 months following penetrating keratoplasty in his right eye aged 23, without developing glaucoma in his surgically naive left eye. Thirty-five eyes (20.6%) from nineteen individuals (16 bilateral and 3 unilateral, 7 male and 12 female patients) remained. Information on whether the glaucoma was primary or secondary to previous ocular surgery was only available in 20 eyes from 12 patients. Sixteen eyes developed glaucoma prior to any surgical intervention, supporting a diagnosis of primary glaucoma. In 2 eyes it was not clear if prior surgery would have influenced the development of glaucoma. (a) Patient 13-I had glaucoma in the right eye diagnosed at 32 years of age and underwent cataract surgery aged 23; additionally, the left eye of the same individual was diagnosed with glaucoma at 31 years of age and was surgically naive until age 36; hence, it is likely that both eyes were predisposed to primary glaucoma. (b) Patient 19-III underwent left eye cataract surgery at 20 years of age and developed glaucoma in the operated eye age 49, although she did not have signs of glaucoma in the unoperated eye at her last visit (age 49), and there is no family history of glaucoma; further, long-term monitoring of this patient may provide more conclusive evidence.

**Figure 4:**
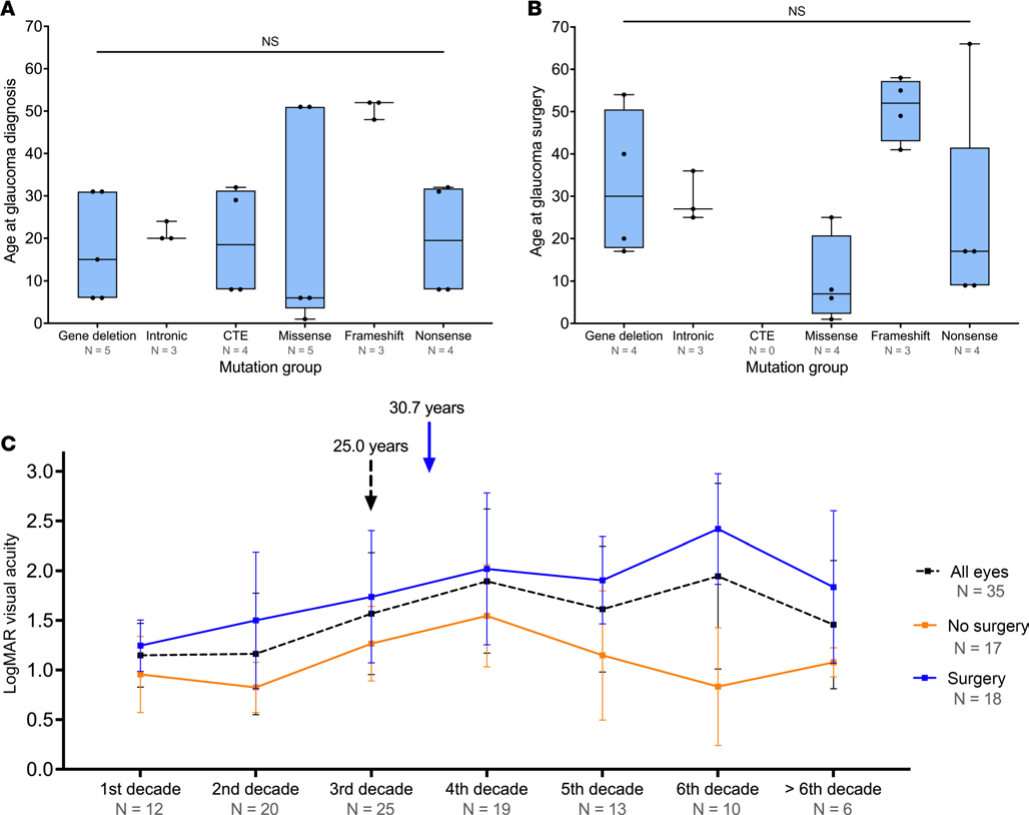
Patients with *PAX6* mutations and glaucoma. Box-and-whisker plots represent differences in (**A**) age at diagnosis and (**B**) age at surgery among mutation groups. The blue box represents the 25th to 75th percentiles. The black line within the box represents the median. Whiskers extend to the minimum and maximum values. All data points were superimposed on the graph. Kruskal-Wallis test was used to compare among mutation groups. (**C**) Line graph demonstrates change in logarithm of the minimum angle of resolution (LogMAR) visual acuity in patients with glaucoma. Data represent mean ± SD. Black dashed arrow and blue arrow represent the mean age at glaucoma diagnosis and surgery, respectively. CTE, C-terminal extension.

Overall, the mean age of glaucoma diagnosis was 25.0 ± 17.3 years. Sex had no effect on the prevalence of glaucoma (Spearman’s *rho* = 0.05, *P =* 0.54). No significant difference in the mean age at glaucoma diagnosis was observed between mutation groups (*P =* 0.22; Table 2 and Figure 4A). The prevalence of glaucoma was significantly different between the individual mutation groups (*P <* 0.001), with gene deletions having a higher prevalence (100%, *Z* score = 4.9), compared with the frameshift mutations with the lowest prevalence (11.4%, *Z* score = –1.6) (Table 2). Glaucoma surgery (including cyclodiode laser, tube surgery, and trabeculectomy) was performed in 18 eyes (51.4%, 6 bilateral and 6 unilateral, 5 male and 7 female patients). The mean age of glaucoma surgery was 30.7 ± 19.5 years. Although patient age at the time of glaucoma operation varied between mutation groups, the differences did not reach statistical significance (*P =* 0.085), likely due to the small number of eyes in the subgroups (Table 2 and Figure 4B). Patients with no surgical intervention for glaucoma maintained a consistently better visual acuity compared with patients who underwent glaucoma surgery (Figure 4C).

### Natural history of ARK in PAX6 variants.

**Figure 5:**
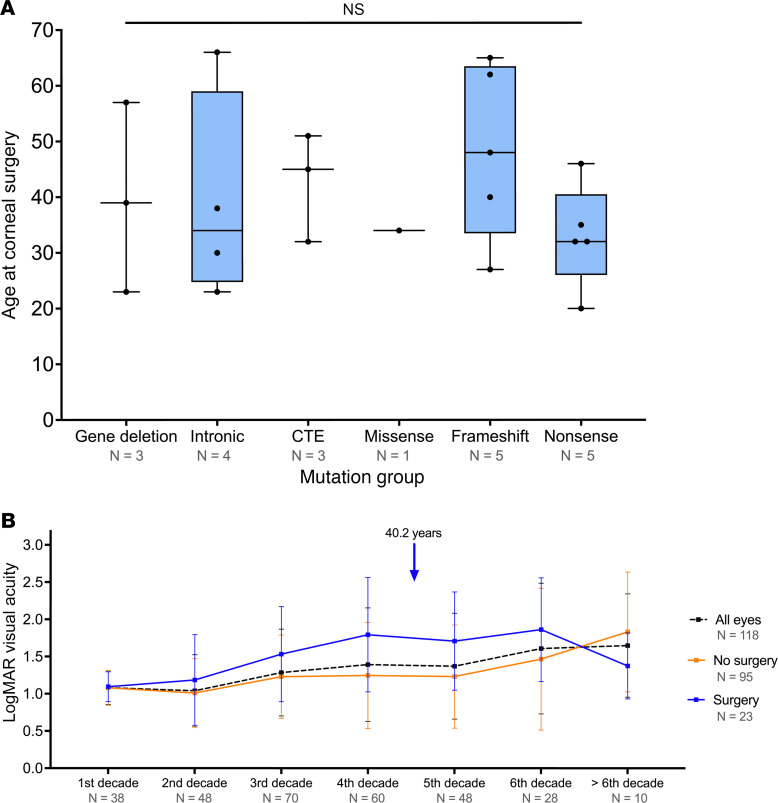
Patients with *PAX6* mutations and aniridia-related keratopathy. (**A**) Box-and-whisker plot represents the difference in the age at corneal surgery among mutation groups. The blue box represents the 25th to 75th percentiles. The black line within the box represents the median. Whiskers extend to the minimum and maximum values. All data points were superimposed on the graph. No statistically significant difference was observed between groups (Kruskal-Wallis test). (**B**) Line graph demonstrates the change in logarithm of the minimum angle of resolution (LogMAR) visual acuity in patients with aniridia-related keratopathy. Data represent mean ± SD. Blue arrow represents the mean age at surgery. CTE, C-terminal extension.

Clinical features of ARK were recorded in 118 eyes (68.6%, 116 bilateral and 2 unilateral, 51 male and 67 female patients), of which 23 eyes had corneal surgery (19.5%, 11 unilateral and 6 bilateral, 8 males and 14 female patients). The mean age of first ARK surgery was 40.2 ± 13.8 years, with no significant difference between mutation subgroups (*P =* 0.68; Figure 5A). The prevalence of ARK was significantly different between the groups (*P =* 0.005), with a higher prevalence in the CTE (*Z* score = 2.1) and nonsense (*Z* score = 2.1) mutations and a lower prevalence in those with missense variants (*Z* score = –3.2; Table 2). Sex had no influence on prevalence (*P =* 0.21). Visual acuity was consistently better in ARK eyes with no surgery from the first decade through to the six decade (Figure 5B).

### Associated ocular and systemic features in patients with PAX6 variants.

**Figure 6:**
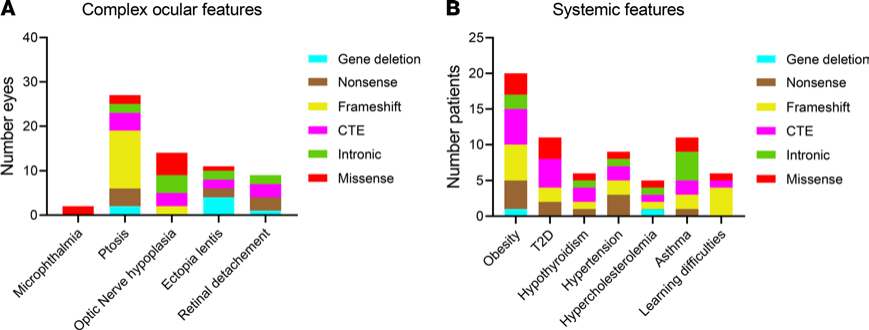
Complex ocular features and systemic features detected in this cohort. (**A**) Distribution of the number of eyes with atypical aniridia or complex ocular phenotypes within the different mutation groups: microphthalmia (*n =* 1), ptosis (*n =* 27), optic nerve hypoplasia (including 1 optic nerve coloboma) (*n =* 14), ectopia lentis (*n =* 11), and retinal detachment (*n =* 9). (**B**) Distribution of systemic features among patients in the different groups: obesity (*n =* 20), type 2 diabetes (T2D) (*n =* 11), hypothyroidism (*n =* 6), hypertension (*n =* 9), hypercholesterolemia (*n =* 4), and asthma (*n =* 11).

Other observed ocular findings among the cohort included ptosis (27 eyes, 15.7%), optic nerve hypoplasia (15 eyes, 8.7%), optic nerve coloboma (1 eye, 0.6%), severe microphthalmia (1 eye, 0.6%), ectopia lentis (11 eyes, 6.4%), and retinal detachment (9 eyes, 5.2%), and evisceration was performed in 2 painful blind eyes with no perception of light from a single patient (patient 1-I) (1.2%; Figure 6A). Ptosis was observed in 14 patients (13 bilateral, 1 unilateral) and was first documented prior to any surgery in 15 eyes, after any form of surgical intervention in 8 eyes, and in 4 eyes with unknown surgical status. No clear correlation with any of the different mutation groups was detected.

Retinal detachment was recorded in 9 eyes (5.2%) from 6 individuals, representing a deletion of the *PAX6* 3′ regulatory region, a nonsense variant (2 unrelated patients with p.[Arg240*]), CTE (p.[Asp413Glufs*112] and p.[*423Leuext*14]), and an intronic mutation (c.357+5G>A). There were no missense or frameshift variants in this cohort (Figure 6A). Of these, 6 occurred in operated eyes, 2 in surgically naive eyes, and the surgical status was unknown for 1 eye.

Several systemic associations were present, including obesity (23.3%, *n =* 20), type 2 diabetes (12.8%, *n =* 11), asthma (12.8%, *n =* 11), hypothyroidism (7%, *n =* 6), and learning difficulties (7%, *n =* 6; Figure 6B), but these were not significantly linked to any mutation group. Other neurological features with lower frequency included autism (2.3%, *n =* 2); central auditory processing disorder, which was diagnosed in 1 patient (1.2%) and suspected in another; and structural brain anomalies, including parietal lobe cavernoma (1.2%, *n =* 1), absent pineal gland (1.2%, *n =* 1), and an abnormality to the posterior aspect of the corpus callosum (1.2%, *n =* 1). Common ocular and systemic features are detailed in Supplemental Table 1.

## Discussion

**Figure 7:**
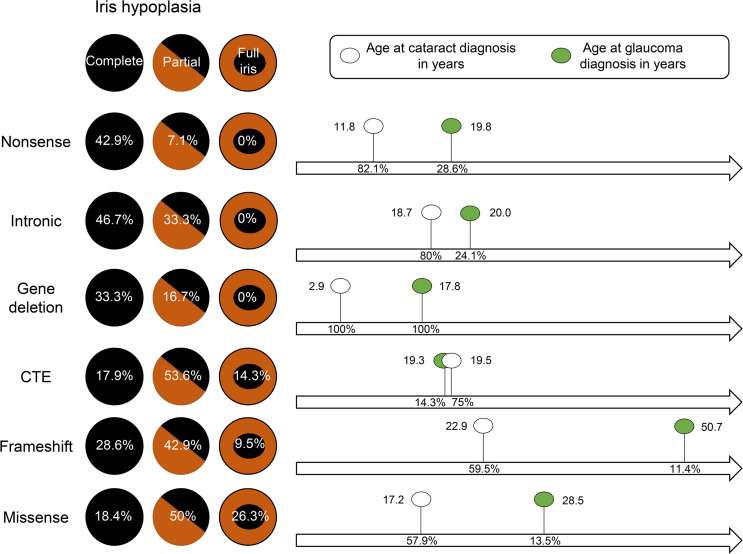
Reference guide for age of cataract and glaucoma development relating to *PAX6* variants. The degree of complete and partial iris hypoplasia versus full iris structure among variant subgroups is shown based in mutation types that included nonsense (*n* = 14), intronic (*n* = 15), gene deletion (*n* = 3), CTE (*n* = 14), frameshift (*n* = 21), and missense (*n* = 19). There is no correlation with iris morphology with the average age at cataract and glaucoma onset. Prevalence of cataracts and glaucoma (in percentages) are shown within arrows. Unknown data are excluded from reference guide.

We present a longitudinal natural history study of patients with aniridia with molecularly confirmed *PAX6* mutations over a mean 16.3-year follow-up. To the best of our knowledge, this is the largest clinically relevant study on genotype-phenotype correlation with respect to systemic associations and ocular prognosis, including long-term visual acuity, cataracts, glaucoma, and ARK in the UK (for a graphical summary see Figure 7).

The majority of *PAX6* variants found in our cohort lead to *PAX6* haploinsufficiency, of which 1 was previously undescribed (patient 23-I) and a further 5 were recently reported by the NHS Wessex Regional Genetics Laboratory, where we sent our patients for diagnostic genetic testing (46). Cross et al. described the genotypes of their *PAX6* cohort and reported the respective phenotypes derived from the genetic request form (46). In contrast, in this study, we present detailed phenotypic descriptions and report significant genotype-phenotype correlations that emerged over time. Missense mutations had the mildest disease course, with increased association of nonaniridia phenotypes, better visual acuity, and lower grades of iris and foveal hypoplasia, but a younger age of surgical intervention for cataract and glaucoma surgery. For those fewer missense patients with more severe iris hypoplasia (grade 5 or 6), the mutations either abolished the *PAX6* start codon (c.2T>G, p.[Met1Arg]) or could in reality affect splicing mechanisms with consequent transcript degradation through nonsense-mediated decay, hence, mimicking loss-of-function variants and leading to classical severe aniridia phenotypes (44, 47). The previously published variant c.372C>A, p.(Asn124Lys) resulted in severe disruption of the PAX6 DNA-binding activity during early eye formation, giving rise to a left microphthalmia and right optic nerve coloboma (23, 48).

A recent analysis of 15 patients with deletions in the *PAX6* 3′ regulatory region showed significantly milder phenotypes compared with all other mutations, with no reported nystagmus, ARK, or foveal hypoplasia (47). We had 1 patient (patient 3-I) with a 3′ UTR gene deletion extending to involve *ELP4* and *DCDC1*; she presented with partial iris hypoplasia, cataracts, glaucoma, and a retinal detachment in a surgically naive eye but no reported ARK.

Previous studies have suggested that patients with PTC and CTE variants have a severe phenotype (3, 49); our data concurred with those findings, with severe deficits in levels of visual acuity. However, findings across these studies demonstrated varying degrees of cataract, glaucoma, and ARK prevalence, and the CTE subgroup trended toward milder grades of both foveal and iris hypoplasia compared with frameshift and nonsense groups. Despite frameshift and nonsense mutations being loss-of-function alleles with the worst visual acuities among operated eyes, each subgroup had different preponderances; for example, nonsense variants resulted in a higher prevalence of cataract, glaucoma, and ARK, whereas frameshift variants resulted in a significantly more severe grade of foveal hypoplasia. These differences may be due to noncoding regulatory elements or other genetic modifiers, which are potentially tissue specific, but further studies with increased number of patients would add evidence toward these phenotypic correlations. Identifying natural history patterns among variant subtypes will help guide clinical trial outcome measures for mutation-specific therapeutics, such as nonsense suppression therapy, which targets nonsense in-frame PTCs.

Clinical features, such as foveal hypoplasia, were observed in three-quarters of patients, and nystagmus was observed in 87.2%, corresponding with reported prevalence rates of 78%–93% and 58%–95%, respectively (3, 7, 39, 47, 50). Previously, the most severe grade of foveal hypoplasia (grade 3 or 4) was associated with PTCs, and milder grades (grades 1 and 2) were associated with gene deletions (51). We found that patients in the frameshift and intronic mutation groups had significantly more severe grades of foveal hypoplasia; no foveal imaging was available for patients in the gene deletion group to assess. Grade of foveal hypoplasia is a predictor of visual acuity (52), a positive correlation between higher foveal hypoplasia grade and worsening visual acuity was demonstrated in our study. Hence, foveal hypoplasia grading may be a useful tool in predicting visual potential in young infants/children and play a role in guiding visual prognosis.

Both operated and unoperated eyes demonstrated a progressive decline in vision with increasing age, but the nonsurgical group maintained a better mean visual acuity for the first 5 decades. Cataracts were the most common ocular comorbidity, with a mean age of surgery of 33.2 ± 14.7 years, in keeping with the reported mean of 20–30 years in the current literature (7, 38, 53, 54). The missense subgroup were operated on earlier (mean age of surgery was 20.8 ± 11.9 years), this is likely due to a better baseline visual acuity and a greater subjective reduction in vision due to the lens opacities. *PAX6* has been identified in cases of congenital cataract (55), and a case series on cataract surgery in patients with congenital aniridia demonstrated some improvement in visual acuity following cataract surgery over a follow-up period of up to 18 months (53). Our study explored the visual acuity changes over an average of 16.3 years in patients with all forms of cataract and found no significant difference in visual acuity among eyes that had undergone cataract surgery (53.7%) compared with eyes with unoperated cataract (46.3%). This may be due to additional progressive glaucomatous changes and ARK, which can also impede visual acuity, despite cataract surgery (38).

The detection rate of glaucoma (20.6%) and the mean age at diagnosis (25.0 ± 17.3 years) were within published time frames, during childhood or early adulthood (7, 28, 56), with prevalence rates between 15% and 66.7% (7, 8, 38, 39, 47, 50, 56). Gramer et al. reported high variability in the age of glaucoma onset in aniridia, with 70% of those diagnosed at between 20 and 69 years of age (56). We found variability in the mean age of glaucoma diagnosis among the mutation groups ranging from 19.3 to 50.7 years of age, but no significant difference in the mean age at diagnosis. Glaucoma surgery (including laser procedures) was performed in 51.4% of patients diagnosed with glaucoma; this is in line with findings of other studies, which have reported between 36% and 71.4% of study populations requiring surgical intervention (7, 28, 38). One study analyzing genotype-phenotype correlations in families with aniridia from Australasia and Southeast Asia identified 11 patients with glaucoma, of which 4 underwent surgical intervention (glaucoma filtration surgery or glaucoma drainage device) at a mean age of 13 ± 5.6 years (7). This departs from our mean age of first surgical intervention at 30.7 ± 19.5 years but may be affected by the differences in study populations. The majority of eyes in our study had primary glaucoma, with only 2 cases having clear secondary glaucoma following surgical intervention. A prospective cases series on congenital aniridia with cataracts reported no incidence of secondary glaucoma following cataract surgery after a mean follow-up postoperatively of 10.2 ± 6.4 months, with a mean patient age at surgery of 25.4 ± 14.77 years (53), but a recent study has reported an overall higher prevalence of secondary glaucoma among patients with ARK requiring advanced corneal surgery compared with patients with ARK treated medically (57).

Published cohort studies have reported the age of ARK onset as between 19 and 33 years of age using differing grading criteria (7, 57). It has also been suggested that a minimal degree of keratopathy is present in all patients with aniridia (6). It is likely that early ARK features are either underreported or go undetected unless specifically investigated, and published standard grading criteria are not always documented in patient clinical notes. Hence, a limitation of this study is that the age of ARK onset was not accurately discernible, and it was not possible to apply ARK grading in this investigation. Although our prevalence rate was 68.6% (118 of 172 eyes), in line with that previously reported (48%–80%; refs. 7, 38, 47, 57), the true rates may be higher. A prospective study would facilitate the acquisition of more detailed information on the development and progression of ARK and its relationship to various surgical interventions. A recent study from Lagali et al. described an association between *PAX6* mutation types and severity of ARK, with whole-gene deletions followed by PTC and CTE variants associated with the most severe grades of ARK. Milder disease forms were in turn associated with missense changes and the mildest forms of ARK in patients with non-*PAX6* variants (6, 58). Accordingly, we found a significantly higher prevalence of ARK in CTE and nonsense mutation subgroups and a significantly lower prevalence with missense mutations as well as the absence of ARK in patient 3-I, with a 3′ UTR gene deletion extending to involve *ELP4* and *DCDC1*. The 2 patients with whole-gene deletion showed presence of ARK with history of corneal surgery, which is consistent with severe ARK diagnosis. In severely affected ARK eyes requiring advanced corneal surgery, a higher rate of glaucoma has been reported in literature (57), which may contribute to worse visual outcomes in the operated group in our study. Conversely, for conservatively managed patients with glaucoma, we observed better visual acuity and reduced need for surgery (seen in the missense subgroup).

Obesity and type 2 diabetes were the most common systemic associations observed in our cohort. Obesity has been reported in both syndromic and nonsyndromic aniridia (7, 28, 36, 59); 23.3% of our patients were affected, but this is below the estimated UK’s general population prevalence of 27.8% (60). Type 2 diabetes, however, was observed in 12.8% of our cohort, which is higher than a previously reported 7% prevalence in a survey of patients with aniridia (28) and is twice the prevalence of diabetes in the UK general population (4.5%–6%) (61, 62). Cases of glucose intolerance, type 1 and 2 diabetes have been reported among patients with aniridia (25, 59, 63, 64), but type 1 diabetes was not observed in our cohort.

The development of type 2 diabetes is multifactorial, with contributions from genetics, epigenetics, lifestyle factors, and obesity (65, 66). One study reported 5 unrelated patients with aniridia, 4 of which had *PAX6* mutations, all with either glucose intolerance or diabetes (63); however, the causal relationship for diabetes remains unclear, as the majority of patients with aniridia do not develop it. Mice with heterozygous *Pax6* variants were shown to have decreased insulin levels (67). Our results suggest that, while obesity may have an effect on the development of diabetes in some patients with aniridia, the presence of *PAX6* mutations is a likely factor. We therefore recommend a low threshold for diabetic assessment to enable clinicians to effectively monitor and modify risk factors to delay the onset or severity in aniridia.

The retrospective data acquired from the medical records and images of patients can pose some limitations, especially relating to accuracy/detail of documentation. The number of clinical visits can also be irregular. Nonetheless, this study has delineated strong genotype-phenotype correlations, which can inform disease prognosis and clinical management decisions. However, phenotypic variability exists within mutation subgroups, suggesting the presence of potential genetic modifiers that require further investigation and/or increased numbers of patients. Prospective studies would help to address limitations, especially in identifying corneal changes in ARK, and to increase data on smaller represented mutation groups, such as gene deletions. In the recent phase 2 ataluren trial for nonsense-mediated aniridia (NCT02647359), change from baseline in maximum reading speed with both eyes was considered the primary outcome measure; secondary outcomes included BCVA in both eyes, critical print size, reading acuity, severity of corneal keratopathy, and iris area measured at 48 weeks. This study can guide the choice of outcome metrics that may be employed for future trials. For example, visual acuity in unoperated eyes remains stable over long periods of follow-up; hence, it is not the optimum clinical trial endpoint, especially for short trials. It also provides insight into the optimum window where intervention and monitoring and early detection may prevent ocular comorbidities and slow disease progression. For future studies, more in depth analysis of prospective corneal changes may provide measurable parameters for patients undergoing treatment for ARK.

In conclusion, we present a natural history evaluation of 86 patients from the UK with molecularly confirmed *PAX6* mutations and describe the phenotypical differences in prevalence, severity, and onset of ocular comorbidities. We provide a valuable tool for clinicians and scientists to aid prognostication, monitoring, and measuring treatment outcomes in individuals with aniridia.

## Methods

### Subjects.

Moorfields Eye Hospital, NHS Foundation Trust, receives secondary and tertiary referrals for patients with congenital aniridia from throughout the UK. Patients who have had genetic testing and received a molecular diagnosis are recorded within a genetics module of the hospital electronic patient record (OpenEyes Electronic Medical Record, Apperta Foundation). In this study, we interrogated the genetics database retrospectively to identify all the families with molecularly confirmed *PAX6* heterozygous mutations on March 1, 2020. Eighty-six patients from sixty-two families with clinically diagnosed isolated aniridia, and *PAX6* heterozygous variants were included in this study. Patients diagnosed with WAGR/WAGRO syndrome were excluded. We established a database to collect phenotyping data from a retrospective review of clinical records and ocular imaging data gathered as part of standard patient care from the first (baseline) clinical visit to their most recent attendance (mean 16.3 ± 12.7 years, range 0.5–61.3 years). The following clinical parameters were recorded; visual acuity (logMAR), iris and foveal morphology using established standard grading scales (see below), ocular and systemic comorbidities (including cataracts, glaucoma, ARK and diabetes), and surgical interventions.

### Molecular screening.

All patients gave informed written consent for genetic testing. Direct Sanger sequencing of the *PAX6* gene (Genbank accession NM_000280.4/ENST00000643871.1 was used for variant nomenclature and exon numbering) was performed at the NHS Wessex Regional Genetics Laboratory and the Rare & Inherited Disease Genomic Laboratory at Great Ormond Street Hospital. Variant analysis, including pathogenicity prediction and novelty, was performed using Alamut Visual v.2.15 (Interactive Biosoftware) and the publicly available Leiden Open Variation Database *PAX6* Mutation Database (http://lsdb.hgu.mrc.ac.uk/home.php?select_db=PAX6). The Wessex Regional Genetics Laboratory recently published all novel *PAX6* variants, including those from patients in our cohort; after which only 1 novel variant remained, and it has been submitted to ClinVar.

### Visual acuity.

BCVA was recorded in logMAR (and converted to this if given in Snellen for statistical analysis). Visual acuity of 1/60 and counting fingers were both recorded as 1.98, whilst hand movements, light perception, and no perception of light was recorded as 2.28, 2.6, and 3.0, respectively (68, 69). Patients with their first baseline visit during infancy often had fixing and following visual acuity. For data analysis, fixing and following were excluded, but the first recorded Snellen or logMAR visual acuity was taken as the first visual acuity recording.

The age at presentation and follow-up periods varied among patients, as reflected by their age and clinical need. Therefore, not all patients had a documented visual acuity for each decade. But where these data were available, 12-month time points were collected and a mean visual acuity for the decade was calculated.

### Iris morphology.

The iris morphology was graded by a single grader. Grading, as described by Grønskov et al. (70), was adapted for this study as follows: no iris abnormality (grade 0); stromal hypoplasia, iris abnormalities, and centrally located pupil (grade 1); stromal hypoplasia, iris abnormalities, and eccentric pupil (grade 2); circumpupillary aplasia with iris abnormalities (grade 3); atypical sector coloboma with less than half circumference absent (grade 4); subtotal iris hypoplasia with more than half circumference absent (grade 5); and complete iris hypoplasia (grade 6). For statistical analysis, grades 1–4 were considered as mild iris hypoplasia, while grades 5 and 6 were classified as severe iris hypoplasia.

### Foveal hypoplasia.

Foveal hypoplasia and nystagmus were identified through clinical notes. Where imaging was available, both Heidelberg Eye Explorer software (Heyex, Heidelberg Engineering) and Topcon IMAGEnet 6 software (Topcon Corp.) were used to assess the degree of foveal hypoplasia in OCT macula scans. Single-line scans were excluded on the basis that the imaging may not have captured the fovea. Volumetric OCT scans of the macula were assessed and the grade of foveal hypoplasia was assigned by a single grader using the grading system described by Thomas et al. (52). The normal foveal structures observed on OCT included the extrusion of the plexiform layers, foveal pit, outer segment lengthening, and outer nuclear layer widening (52). Grade 1 foveal hypoplasia was assigned when extrusion of the plexiform layers was absent and all other foveal features were present; grade 2 hypoplasia included features of grade 1 and the absence of the foveal pit; grade 3 included features of grade 2 and the absence of outer segment lengthening; and in grade 4, all foveal features were absent (52). For statistical analysis, grades 1 and 2 were considered as mild foveal hypoplasia, while grades 3 and 4 were regarded as severe.

### Ocular comorbidity and surgical intervention.

For individuals who developed glaucoma, the date of onset was taken as the commencement of intraocular pressure–lowering drugs; those with ocular hypertension or who developed secondary glaucoma were excluded. For age of onset, patients with cataracts or glaucoma on their first baseline visit were excluded, unless they were at or below 1 year of age, as the date of onset could not be accurately ascertained. All procedures requiring patient consent were recorded as a form of surgical intervention, including all types of cataract extraction; glaucoma laser treatment, tube surgery, and trabeculectomy; and ARK-related superficial keratectomy, corneal grafts, limbal stem cell transplants, and keratoprosthesis. Aniridic fibrosis syndrome is a rare complication seen in aniridia and it was not identified in our cohort.

### Statistics.

Statistical analysis was performed using Microsoft Excel 2013 and SPSS v. 26 (IBM). Continuous data were presented as population mean ± SD, while categorical data were presented in frequency and/or percentage. Kruskal-Wallis test (1-way analysis of variance on ranks) was used to compare continuous variables across mutation groups. When a statistically significant difference was detected, Dunn’s post hoc test was used to assess pairwise comparisons. Contingency tables, along with the χ^2^ test or likelihood ratio, were used to compare categorical variables (2-tailed testing). Adjusted standardized residuals (*Z* score) were used as a post hoc test. Cells with a *Z* score absolute value larger than 1.96 were considered statistically significant. Spearman’s signed-rank correlation coefficient (*rho*) was used to investigate correlation between foveal hypoplasia and visual acuity. *P* values of less than 0.05 were considered statistically significant.

### Study approval.

This study was approved by Moorfields Eye Hospital and the National Research Ethics Committee (REC12/LO/0141); it was conducted in adherence to the tenets of the Declaration of Helsinki. Informed written consent was obtained from all participants.

## Author contributions

MM supervised the research. VK acquired the data. VK, AMH, and DLC analyzed the data. VK, AMH, DLC, and MM wrote the manuscript.

## Supplementary Material

Supplemental data

## Figures and Tables

**Table 1:**
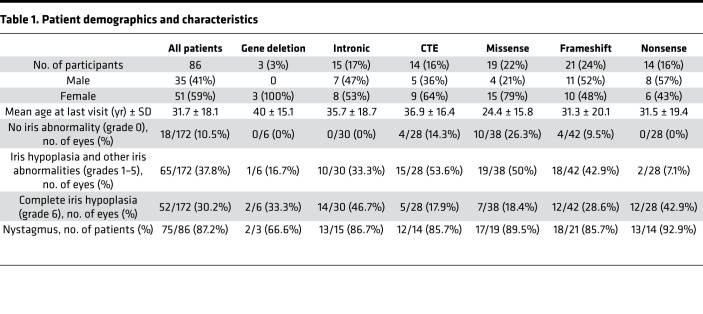
Patient demographics and characteristics

**Table 2:**
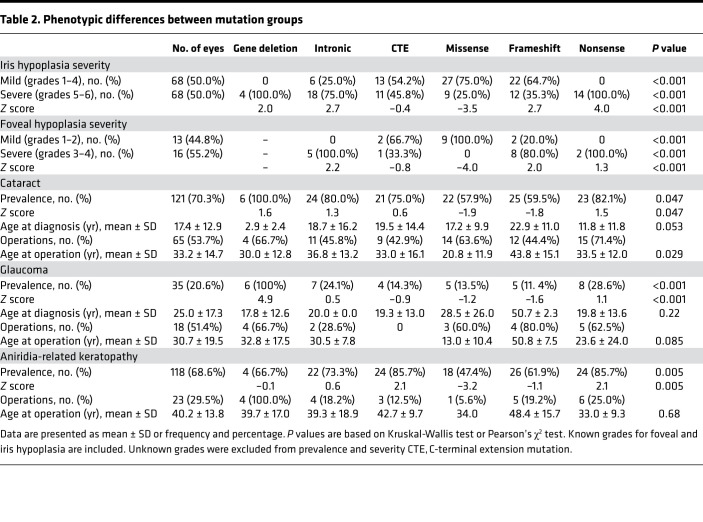
Phenotypic differences between mutation groups

## Version 1 06/08/2021

In-Press Preview

## Version 2 07/22/2021

Electronic publication

## References

[1] Moosajee M, et al. PAX6-Related Aniridia. In: Adam MP, Ardinger HH, Pagon RA, et al, eds. *GeneReviews((R))*. University of Washington; 1993. 20301534

[2] Hingorani M (2012). Aniridia. Eur J Hum Genet.

[3] Hingorani M (2009). Detailed ophthalmologic evaluation of 43 individuals with PAX6 mutations. Invest Ophthalmol Vis Sci.

[4] Calvão-Pires P (2014). Congenital aniridia: clinic, genetics, therapeutics, and prognosis. Int Sch Res Notices.

[5] Lim HT (2017). PAX6 aniridia syndrome: clinics, genetics, and therapeutics. Curr Opin Ophthalmol.

[6] Lagali N (2020). Early phenotypic features of aniridia-associated keratopathy and association with PAX6 coding mutations. Ocul Surf.

[7] Souzeau E (2018). *PAX6* molecular analysis and genotype-phenotype correlations in families with aniridia from Australasia and Southeast Asia. Mol Vis.

[8] Yokoi T (2016). Genotype-phenotype correlation of PAX6 gene mutations in aniridia. Hum Genome Var.

[9] Jordan T (1992). The human PAX6 gene is mutated in two patients with aniridia. Nat Genet.

[10] Hanson IM (1994). Mutations at the PAX6 locus are found in heterogeneous anterior segment malformations including Peters’ anomaly. Nat Genet.

[11] Shaham O (2012). Pax6: a multi-level regulator of ocular development. Prog Retin Eye Res.

[12] Stanescu D (2007). Continuous expression of the homeobox gene Pax6 in the ageing human retina. Eye (Lond).

[13] Vincent MC (2003). Screening for PAX6 gene mutations is consistent with haploinsufficiency as the main mechanism leading to various ocular defects. Eur J Hum Genet.

[14] Kokotas H, Petersen MB (2010). Clinical and molecular aspects of aniridia. Clin Genet.

[15] Glaser T (1992). Genomic structure, evolutionary conservation and aniridia mutations in the human PAX6 gene. Nat Genet.

[16] Davis A, Cowell JK (1993). Mutations in the PAX6 gene in patients with hereditary aniridia. Hum Mol Genet.

[17] Wolf MT (1998). Ten novel mutations found in Aniridia. Hum Mutat.

[18] Lima Cunha D (2019). The spectrum of PAX6 mutations and genotype-phenotype correlations in the eye. Genes (Basel).

[19] Lauderdale JD (2000). 3′ deletions cause aniridia by preventing PAX6 gene expression. Proc Natl Acad Sci U S A.

[20] Bobilev AM (2016). Assessment of PAX6 alleles in 66 families with aniridia. Clin Genet.

[21] Robinson DO (2008). Genetic analysis of chromosome 11p13 and the PAX6 gene in a series of 125 cases referred with aniridia. Am J Med Genet A.

[22] Tzoulaki I (2005). PAX6 mutations: genotype-phenotype correlations. BMC Genet.

[23] Williamson KA (2020). Recurrent heterozygous PAX6 missense variants cause severe bilateral microphthalmia via predictable effects on DNA-protein interaction. Genet Med.

[24] Redeker EJ (2008). Multiplex ligation-dependent probe amplification (MLPA) enhances the molecular diagnosis of aniridia and related disorders. Mol Vis.

[25] Motoda S (2019). Case of a novel PAX6 mutation with aniridia and insulin-dependent diabetes mellitus. J Diabetes Investig.

[26] Nishi M (2005). A case of novel de novo paired box gene 6 (PAX6) mutation with early-onset diabetes mellitus and aniridia. Diabet Med.

[27] Panneerselvam A (2019). PAX proteins and their role in pancreas. Diabetes Res Clin Pract.

[28] Netland PA (2011). Ocular and systemic findings in a survey of aniridia subjects. J AAPOS.

[29] Abouzeid H (2009). PAX6 aniridia and interhemispheric brain anomalies. Mol Vis.

[30] Kikkawa T (2019). The role of Pax6 in brain development and its impact on pathogenesis of autism spectrum disorder. Brain Res.

[31] Dansault A (2007). Three new PAX6 mutations including one causing an unusual ophthalmic phenotype associated with neurodevelopmental abnormalities. Mol Vis.

[32] Sisodiya SM (2001). PAX6 haploinsufficiency causes cerebral malformation and olfactory dysfunction in humans. Nat Genet.

[33] Chien YH (2009). Eye anomalies and neurological manifestations in patients with PAX6 mutations. Mol Vis.

[34] Mitchell TN (2003). Polymicrogyria and absence of pineal gland due to PAX6 mutation. Ann Neurol.

[35] Heyman I (1999). Psychiatric disorder and cognitive function in a family with an inherited novel mutation of the developmental control gene PAX6. Psychiatr Genet.

[36] Ferreira MAT (2019). WAGRO syndrome: a rare genetic condition associated with aniridia and additional ophthalmologic abnormalities. Arq Bras Oftalmol.

[37] Dubey SK (2015). Mutational analysis and genotype-phenotype correlations in southern Indian patients with sporadic and familial aniridia. Mol Vis.

[38] Park SH (2010). Clinical features of Korean patients with congenital aniridia. Korean J Ophthalmol.

[39] You B (2020). Mutation spectrum of *PAX6* and clinical findings in 95 Chinese patients with aniridia. Mol Vis.

[40] Zhang X (2011). Mutation spectrum of PAX6 in Chinese patients with aniridia. Mol Vis.

[41] Park SH (2012). Molecular analysis of the PAX6 gene for congenital aniridia in the Korean population: identification of four novel mutations. Mol Vis.

[42] Sapna S (2008). Elliptical anterior iris stromal defects associated with PAX6 gene sequence changes. J AAPOS.

[43] Hever AM (2006). Developmental malformations of the eye: the role of PAX6, SOX2 and OTX2. Clin Genet.

[44] Filatova AY (2019). Functional reassessment of PAX6 single nucleotide variants by in vitro splicing assay. Eur J Hum Genet.

[45] Tarilonte M Activation of cryptic donor splice sites by non-coding and coding PAX6 variants contributes to congenital aniridia. J Med Genet.

[46] Cross E (2020). Screening of a large PAX6 cohort identified many novel variants and emphasises the importance of the paired and homeobox domains. Eur J Med Genet.

[47] Vasilyeva TA (2020). Analysis of genotype-phenotype correlations in PAX6-associated aniridia. J Med Genet.

[48] Lee S (2020). Impaired DNA-binding affinity of novel PAX6 mutations. Sci Rep.

[49] Aggarwal S (2011). Run-on mutation in the PAX6 gene and chorioretinal degeneration in autosomal dominant aniridia. Mol Vis.

[50] Sannan NS (2017). Correlation of novel PAX6 gene abnormalities in aniridia and clinical presentation. Can J Ophthalmol.

[51] Pedersen HR (2020). PAX6 genotypic and retinal phenotypic characterization in congenital aniridia. Invest Ophthalmol Vis Sci.

[52] Thomas MG (2011). Structural grading of foveal hypoplasia using spectral-domain optical coherence tomography a predictor of visual acuity?. Ophthalmology.

[53] Wang JD (2017). Congenital aniridia with cataract: case series. BMC Ophthalmol.

[54] Shiple D (2015). Keratopathy, cataract, and dry eye in a survey of aniridia subjects. Clin Ophthalmol.

[55] Ma AS (2016). Sporadic and familial congenital cataracts: mutational spectrum and new diagnoses using next-generation sequencing. Hum Mutat.

[56] Gramer E (2012). Glaucoma and frequency of ocular and general diseases in 30 patients with aniridia: a clinical study. Eur J Ophthalmol.

[57] Yazdanpanah G (2020). Management of congenital aniridia-associated keratopathy: long-term outcomes from a tertiary referral center. Am J Ophthalmol.

[58] Voskresenskaya A (2017). Clinical and morphological manifestations of aniridia-associated keratopathy on anterior segment optical coherence tomography and in vivo confocal microscopy. Ocul Surf.

[59] Boese EA (2020). Novel intragenic *PAX6* deletion in a pedigree with aniridia, morbid obesity, and diabetes. Curr Eye Res.

[60] Scheelbeek PFD (2019). Potential impact on prevalence of obesity in the UK of a 20% price increase in high sugar snacks: modelling study. BMJ.

[61] Holman N (2015). Current prevalence of Type 1 and Type 2 diabetes in adults and children in the UK. Diabet Med.

[62] Diabetes Digital Media. Diabetes Prevalence. https://www.diabetes.co.uk/diabetes-prevalence.html Updated January 15, 2019. Accessed June 9, 2021

[63] Yasuda T (2002). PAX6 mutation as a genetic factor common to aniridia and glucose intolerance. Diabetes.

[64] Peter NM (2013). PAX6 mutation in association with ptosis, cataract, iris hypoplasia, corneal opacification and diabetes: a new variant of familial aniridia?. Clin Exp Ophthalmol.

[65] Malone JI, Hansen BC (2019). Does obesity cause type 2 diabetes mellitus (T2DM)? Or is it the opposite?. Pediatr Diabetes.

[66] Zheng Y (2018). Global aetiology and epidemiology of type 2 diabetes mellitus and its complications. Nat Rev Endocrinol.

[67] Mitchell RK (2017). The transcription factor *Pax6* is required for pancreatic β cell identity, glucose-regulated ATP synthesis, and Ca^2+^ dynamics in adult mice. J Biol Chem.

[68] Lange C (2009). Resolving the clinical acuity categories “hand motion” and “counting fingers” using the Freiburg Visual Acuity Test (FrACT). Graefes Arch Clin Exp Ophthalmol.

[69] Schulze-Bonsel K (2006). Visual acuities “hand motion” and “counting fingers” can be quantified with the freiburg visual acuity test. Invest Ophthalmol Vis Sci.

[70] Grønskov K (1999). Mutational analysis of PAX6: 16 novel mutations including 5 missense mutations with a mild aniridia phenotype. Eur J Hum Genet.

